# Department for Education Statutory Guidance for Relationships and Sex Education in England: A Rights-Based Approach?

**DOI:** 10.1007/s10508-022-02340-5

**Published:** 2022-09-28

**Authors:** Emily Setty, Emma Dobson

**Affiliations:** 1 Department of Sociology, University of Surrey, 11 AD 03, Guildford, GU2 7XH Surrey UK; 2 School of Education, University of Durham, Durham, UK

**Keywords:** Relationships and sex education, Children and Social Work Act (UK), Department for Education (England), Young people, Sex education

## Abstract

In England, the Children and Social Work Act (HMSO, 2017) bestowed compulsory status on relationships and sex education (RSE), which means that young people’s right to receive RSE has been codified in law. This paper analyzes how this right is upheld and enacted within the Department for Education (DfE) (2019) statutory guidance on RSE for schools in England. The analysis suggests that the guidance features contradictory discourses in which young people’s rights are ostensibly advanced, but remain structured by adult-centric, heteronormative understandings of sex and relationships. It upholds a decontextualized and legalistic approach to rights, responsibilities, informed choice, and decision making. A narrow conception of rights is particularly evident regarding young people’s digital sexual cultures, which are predominantly framed in terms of risk and harm. We argue that scholars should investigate how educators are designing and delivering RSE in light of the guidance, and the opportunities for and obstacles to a genuinely “rights-based” approach to RSE. While the policy discussed in this article is specific to England, the discussion has wider relevance for practitioners and policymakers across cultural and geographic contexts as it draws upon a model for analyzing how young people’s sexuality is presented and addressed in legislative and curricular documentation.

## Introduction

Determining how to deliver relationships and sex education (RSE) according to “rights-based” principles is subject to current debate within the field. Rights-based RSE is a contested concept (Moore, 2013). It has been variously conceived of as the right to receive RSE and the right to sexual health and well-being, as well as in terms of the teaching approach and pedagogical style that frames the delivery of RSE content.

In England, the Children and Social Work Act (2017) bestowed compulsory status upon RSE, suggesting that RSE is rights-based in as much as young people now have a legal right to receive RSE. Prior to this legislative change, RSE provision in England was patchy and inconsistent across schools and local authority areas, with schools having a legal duty only to provide a limited curriculum covering the “science” of sex, reproduction, and puberty (e.g., British Humanist Association, 2017; Emmerson, 2018; National Children’s Bureau, 2016; Ofsted, 2013). How the newly codified right to RSE is envisaged and enacted within the Department for Education (DfE) Statutory guidance on RSE for schools in England (DfE 2019) requires further exploration, however, to understand the conceptualization of rights-based RSE that it presents. Is the guidance rights-based in its acknowledgment of young people’s reciprocal and relational right to sexual health and well-being? Can it support teachers in delivering RSE that utilizes a rights-based approach?

In this paper, we first examine the extent to which different models of RSE align with conceptualizations of rights-based RSE and consider what “rights-based” provision may look like. We then analyze the DfE (2019) guidance, exploring the conception of rights that it explicitly and implicitly endorses. We engage in close reading of the manifest and latent content of the guidance, focusing specifically on outcomes to be achieved in secondary schools, as this setting is identified as where pupils should receive full education covering “intimate relationships and sex” (DfE, 2019, p. 4). While the policy discussed in this article is specific to England, the discussion has wider relevance for practitioners and policymakers across cultural and geographic contexts as it suggests a model to analyze how young people’s sexuality is presented and addressed in legislative and curricular documentation.

### Youth Sexuality as a Human Right

As in other countries, RSE policy and practice in England has moved steadily “toward compliance with a rights-based framework” (Yilmaz & Willis, 2020, p. 12). It has evolved from an earlier emphasis on health, hygiene, biology, and reproduction, to more holistic education focused on sex, sexuality, and relationships, typically referred to as comprehensive sex education (CSE) (Pilcher, 2005). The receipt of CSE is identified as a human right by the United Nations Convention on the Right of the Child (UNCRC), the Joint United Nations Programme on HIV/AIDS (UNAIDS), the United Nations Educational, Scientific and Cultural Organization (UNESCO), the United Nations Population Fund (UNFPA), and the World Health Organization (WHO), all of which recognize and reinforce young people’s sexual rights (Blake & Aggleton, 2017). There is recognition of young people’s evolving capacity to exercise sexual rights on their own behalf, balanced with a need for protection and guidance to support their path to healthy adulthood (Berglas et al., 2014a, 2014b, p. 288). In 2012, the UN Commission on Population and Development reaffirmed the connection between the principles of sexual rights for young people and gender equality in Commission on Population and Development 2012/1: Adolescents and Youth (Berglas et al., 2014a, 2014b).

It is suggested that teaching and informing young people about their rights is beneficial for skill development and helps prepare young people for participation as productive citizens in a democratic society (Goldman, 2008; Levesque, 2000). RSE is, therefore, often advanced as a “public good.” Given that conceptualizations of what is “good” and “appropriate” for young people remain contested, however, so too does the nature of RSE (Moore, 2013). Different forms of RSE thus remain despite a broad consensus that young people have a right to education about sex and relationships.

### Approaches to Relationships and Sex Education

RSE may take the following broad forms (Yankah, 2016): abstinence-based sex education; the above-mentioned CSE; and holistic sex education (HSE). Underpinning these approaches are different narratives about sex, sexuality, and relationships that shape their alignment to conceptualizations of rights (Irvine, 2002; Jones, 2011). Advocates of liberal models of RSE—CSE and HSE—oftentimes present them as inherently superior to abstinence-based RSE (see Rasmussen, 2012) and as concerned with universal values of individual freedom and rights to self-determination within a liberal democracy (see Steutal & Siecker, 2004). It is argued that abstinence-based RSE is, in contrast, based on a conservative outlook that seeks to impart normative facts about sex, sexuality, and relationships which limit the range of legitimized sexual expression and identity, and afford little agency to learners (Johnson, 1996; Jones, 2011; Levesque, 2000).

Connell and Elliot (2009) explain that abstinence-based RSE rests upon a construction of childhood innocence that requires protection from “corrupting” forces. They argue that ideas of purity and vulnerability perpetuate class, gender, sexuality, and race-based inequalities with the aim of preserving heteronormative ideals about the sanctity of family life (also see Egan & Hawkes, 2007; Johnson, 1996). Abstinence-based RSE has, therefore, been described as denying young people the right to RSE as they are simply instructed to abstain from sexual activity, or as involving a very limited acknowledge of rights. It prevails in parts of the USA, but is seen more widely in response to contemporary issues such as digital sexual culture.

CSE and HSE instead conceive of young people as having a right to full information about sex, sexuality, and relationships. Both promote skills development and active participation by learners. CSE tends to be advanced on pragmatic or public health grounds, in which information and guidance is intended to support informed decision making, most often to avoid negative sexual health outcomes (Jones, 2011; Midema et al., 2020; Ponzetti, 2016). It has been conceptualized as “abstinence-plus” education because oftentimes the message is to abstain but if not, to practice sex safely (Midema et al., 2020). By contrast, HSE more fully engages with both the risks and pleasures of sexuality beyond a harm reduction paradigm (Midema et al., 2020; Parker et al., 2004). Here, young people are conceived of as having a right to sex, sexuality, and relationships as goods in and of themselves, as well as protection from harm (Jones, 2011; Ketting & Winkelmann, 2013; Midema et al., 2020; Moore, 2013). Despite these conceptual distinctions drawn in the literature, there are some overlaps in the application of CSE and HSE. In some areas, for example, in Canada (see SIECCAN, 2019) CSE is not considered “abstinence-plus” but is framed in the same way as HSE has been conceptualized. CSE is also defined more broadly in UNESCO’s technical guidance on RSE (UNESCO, 2018).

Young people’s access to discourses of positive rights to sex, sexuality, and relationships can be limited and has been subject to ongoing debate and resistance (Jackson & Scott, 2010; Moore, 2013). Narrow conceptualizations of youth sexuality can homogenize and pathologize young people’s developing sexual subjectivities and experiences and deny their agency which precludes full acknowledgment of positive rights (Brennan & Epp, 2015; Epstein et al., 2012). This has implications for the extent to which RSE is rights-based in terms of going beyond risk and harm to support rights to sexual health and well-being.

Moreover, critical scholars argue that rights—be they positive or negative—unfold within a sociocultural context and RSE needs to account for the inequalities and injustices that shape sex, sexuality, and relationships and that have implications for rights (Connell & Elliot, 2009; Helmich, 2009; Johnson, 1996; Jones, 2011; Ketting & Winkelmann, 2013; Levesque, 2000). These scholars caution that if RSE does not speak to the norms and inequalities that marginalize groups, it may become normalizing and harmful (Elia & Eliason, 2010; Jones, 2011; Levesque, 2000; Midema et al., 2020). Parker et al., (2004, p. 388) contend that RSE should instead uphold “a more liberating and celebratory concept of sexual rights as part of a broader emancipation of the social, as well as the sexual world.” This involves “teaching children sexual agency coupled with an inclusive, social justice-informed perspective” (Elliot & Connell, 2009, p. 96). These links between sexual rights and substantive equity are reflected in international human rights frameworks that construct RSE in terms of sexual and gender equality (Mayo, 2011). Here, young people’s agency and sexual subjectivity, and, by association, their rights, are conceived of relational and embedded in social contexts (Cense, 2019).

### Defining Rights-Based Relationships and Sex Education

The range of approaches to RSE and the different levels and forms of sexual autonomy that they confer upon young people means that the interpretation of international human rights frameworks into local and national RSE policy is complex and will be greatly influenced by the context in which it operates. While there is no consensus on the definition of rights-based RSE, a review of American policy documents and practitioner perspectives conducted by Berglas et al. (2014b) identified four underlying themes or core elements of rights-based RSE (Table 1). These principles are repeated in rights-based “standards, guidelines and program materials” used across different countries and contexts, “lending support to the validity of a conceptual definition” (Berglas et al., 2014b, p. 69) and offering a measure of rights-based provision that can be used to evaluate DfE guidance.

**Table 1:** Principles of Rights-Based Sex Education (according to Berglas et al., 2014a, 2014b)

Principle	Definition
Acknowledge the sexual rights of youth	Youth have inalienable rights, expressed in international human rights law that must be accounted for when considering access to and content of sexual education (p.65). This includes a right to self-determination, including the rights to express their sexuality, decide whether and when to engage in sex, choose whether and when to have children and pursue a safe and pleasurable sexual life. It puts sexual rights in the hands of youth and aims to instil knowledge, skills, and agency, so that young people can determine and voice their own needs while also understanding their corresponding responsibility to respect the rights of others (p. 65)
Aim to increase well-being rather than focus on risk-avoidance	Expansion of programmatic goals beyond the current emphasis on discouraging sex outside of marriage or preventing unintended pregnancy or STDs… to affect other realms of wellbeing (pp. 65–66). It aims to achieve broader goals related to empowerment, sexual assertiveness, expectations and even civic engagement (p. 66)
Address contextual issues that influence decision making	Broad in program content, moving beyond an emphasis on prevention of pregnancy and disease… to address larger contextual issues that affect adolescents’ sexual decision making (p. 66)
Adopt a participatory approach	Discarding more didactic models of delivery in favour of methods that are participatory, interactive and youth-centred (p. 66)

Despite the review being conducted in America, there are clear parallels with the English context. For example, the identification of barriers to implementation of a rights-based approach mirror identified issues hindering the provision of RSE in England, including balancing youth and parental rights, teachers’ difficulties in facilitating open and bi-directional discussion about complex issues, debates around topic inclusion, and reluctance to be “sex positive” instead of or in addition to focusing on harm reduction. The introduction of mandatory RSE in schools in England offers the opportunity to examine whether and how the principles identified by Berglas et al. (2014b) are reflected in the DfE (2019) guidance and how these principles may be applied within the RSE curriculum and classroom.

### Assessing the Relationships and Sex Education Guidance in England

By codifying young people’s right to RSE in law, it could be concluded that current policy in England successfully meets the conceptualization of rights-based RSE as defined by a right to education. At issue, however, is who gets access to RSE; how young people’s developing sexualities and sexual subjectivities are positioned within RSE; the extent to which this positioning is inclusive and considerate of the contextual and societal influences in young people’s lives; and how this expands their understanding of their rights and the rights of others to experience sexual health, pleasure, and well-being free from prejudice and discrimination. The analysis of the DfE (2019) statutory guidance was conducted with the aim of identifying the government’s stance on these issues based upon how these rights are upheld and enacted within the guidance. In so doing, we offer an overall judgment on the extent to which the guidance is rights-based.

When assessing the guidance, we used the model of rights-based RSE suggested by Berglas et al. (2014a) and were guided by a priori codes to identify and analyze both the explicit (manifest) and implicit (latent) content of the guidance. These codes included:

- The rationale or purpose for the provision of RSE;
- The framing of childhood and youth in terms of sex, relationships, and sexuality;
- The framing of rights (individual, relational, contextual, and/or critical); and
- The positioning of the learner and the teacher within the pedagogic process and their roles in identifying the issues to be addressed.

The preamble and explanatory sections (pp. 4–18) of the guidance and learning outcomes for secondary school pupils (pp. 25–30) were subject to analysis. The analytical process involved a thorough reading of the guidance by both authors. Each author then independently coded the guidance. The codes were initially descriptive and referred, for example, to the specific area of sex and relationships or the stated aim of RSE. Each author then organized the codes into categories and cross-checked one another’s codes and categories. The authors discussed and mutually resolved any discrepancies. The authors then collaboratively examined each category to identify themes pertaining to the extent to which and how young people’s rights were advanced across the guidance. The analytical process was supported by memos produced by each author during each stage of the analysis, in which initial interpretations were recorded. The themes were developed in relation to the wider theoretical and conceptual literature on RSE.

## Findings

This analysis firstly examines the direct engagement with young people’s sexual rights within the guidance and the model of rights this aligns with and then explores the extent to which the guidance follows the model of rights-based education proposed by Berglas et al. (2014a) through identifying its underlying principle, programmatic goals, content, and pedagogy. The themes identified in the guidance are presented in terms of each aspect of Berglas et al. (2014a) model.

### Young People’s Sexual Rights

Within the preamble of the guidance, there is a positive conceptualization of sexual health and well-being and extensive commitment to upholding young people’s rights to RSE. The guidance appears to take a fundamentally liberal approach to RSE, advocating that:

“To embrace the challenges of creating a happy and successful adult life, pupils need knowledge that will enable them to make informed decisions about their wellbeing, health and relationships and to build their self-efficacy. Pupils can also put this knowledge into practice as they develop the capacity to make sound decisions when facing risks, challenges and complex contexts” (DfE, 2019, p. 8).

There is emphasis placed on the contexts in which sex, sexuality, and relationships occur, and the importance of equality and diversity:

“Pupils are to know…how stereotypes, in particular stereotypes based on sex, gender, religion, sexual orientation or disability, can cause damage [and]… that in school and in wider society they can expect to be treated with respect by others, and that in turn they should show due respect to others, including people in positions of authority and due tolerance of other people’s beliefs” (DfE, 2019, p. 28).

In upholding these rights, the guidance advocates making connections between RSE and the wider curriculum, in a “whole school approach” that covers school rules, policies, and interventions (DfE, 2019, p. 40).

“Schools should be alive to issues such as everyday sexism, misogyny, homophobia and gender stereotypes and take positive action to build a culture where these are not tolerated, and any occurrences are identified and tackled. Staff have an important role to play in modelling positive behaviours. School pastoral and behaviour policies should support all pupils” (DfE 2019, p. 14).

The guidance acknowledges that young people have the freedom to hold and express “diverse opinions within the law” (DfE, 2019, p. 26) and commits to upholding these rights in both online and offline contexts, stating that the standards for rights and responsibilities apply equally in these contexts. It states that young people have a right to information and knowledge and recognizes their rights to make personal decisions, while highlighting the importance of respecting the decisions of others. This includes where those rights are codified in law (e.g., regarding violent, abusive, and harassing behavior). This consideration of individual rights and the rights of others suggests a reciprocal model of rights:

“Pupils should be well informed about the full range of perspective and, within the law, should be well equipped to make decisions for themselves about how to live their own lives, whilst respecting the right of others to make their own decisions and hold their own beliefs” (DfE, 2019, p. 26).

Despite this, there is a lack of explicit attention to the conditions in which these rights and responsibilities unfold or may be delimited in practice, an issue examined further in the next sections of the analysis.

### Underlying Principle

The guidance states that RSE should support free and informed choice and decision making to: “give them [pupils] the knowledge and capability to take care of themselves and receive support if problems arise” (DfE, 2019, p. 4). This is to include the provision of “facts” about the different “choices” connected to sex and relationships and skills development, stating that “[t]eaching will include well-chosen opportunities and contexts for pupils to embed new knowledge so that it can be used confidently in real life situations” (DfE, 2019, p. 8). A learning outcome pertaining to “Respectful relationships,” for example, includes knowledge of “…practical steps they can take in a range of different contexts to improve or support respectful relationships” (DfE, 2019, p. 27).

Running alongside this narrative of choice and decision making is a legalistic approach to rights and responsibilities (DfE, 2019). Throughout the guidance, the law is brought to the fore.

“It is recognised that there will be a range of opinions regarding RSE. The starting principle when teaching each of these must be that the applicable law should be taught in a factual way so that pupils are clear on their rights and responsibilities as citizens” (DfE, 2019, p. 26).

Upholding the law is the most immediate and obvious underlying principle of the guidance. The term “law” or “unlawful” appears 19 times in the guidance, while the term “legal” or “illegal” is mentioned 18 times. Within the five pages of learning outcomes, there are seven statements referring to legal guidance, legislation, or legal threats such as “severe penalties” and “jail.” Adherence to the law is embedded within each of the learning outcomes, with a page dedicated to how teachers can apply a legal focus to taught material (DfE, 2019, p. 30).

“In all schools, teaching should reflect the law (including the Equality Act 2010) as it applies to relationships, so that young people clearly understand what the law allows and does not allow, and the wider legal implications of the decisions they make” (DfE, 2019, p. 13).

This quote also demonstrates that the stated commitment to equality is framed primarily in terms of the law. There are no specifics regarding how equality and diversity issues apply to the different topics, or ways of thinking about equality and diversity beyond the law. There is no mention of rights nor of skills development in advocating for one’s (or another’s) rights.

More specifically, while schools are told to “comply with the requirements of the Equality Act 2010” (DfE, 2019, p. 3) and “marriage and civil partnership” is noted as a protected characteristic (DfE, 2019, p. 13), the guidance privileges teaching about marriage (with a footnote stating that this is to include civil partnerships). There is little to suggest that *not* entering into marriage or civil partnership is a legitimate choice. Sex and relationships are framed in terms of commitment and married family life, connected to “human happiness” and “bringing up children” (DfE, 2019, p. 27). “Stable relationships” (DfE, 2019, p. 27) are, therefore, presented as a self-evident goal, another underlying principle of RSE guidance.

“Families” are discussed in terms of committed relationships (with marriage/civil partnership the assumed ideal) with an emphasis on “…the roles and responsibilities of parents… [and] the characteristics of successful parenting” (DfE, 2019, p. 27). This is presented as value-neutral, but there is no discussion of diverse family types. The guidance also states that “Pupils are to be taught the facts and the law about sex, sexuality, sexual health and gender identity in an age-appropriate and inclusive way” (DfE, 2019, p. 26). While appearing neutral through the words “facts” and “law,” there are implicit value systems at play. For example, it states that RSE should involve “…an equal opportunity to explore the features of stable and healthy same-sex relationships” (DfE, 2019, p. 26). This meets the requirement of the Equality Act 2010 not to discriminate based on sexual orientation yet assumes that what it means for relationships to be stable and healthy is known, agreed upon and aspired to.

There is, furthermore, an individualistic emphasis on equipping pupils to identify whether others are “trustworthy… unsafe… and, how to seek help or advice” (DfE, 2019, p. 27). While the guidance posits that rights and responsibilities online are the same as offline, this is contradicted by the learning outcomes. For example, it states that pupils are to be taught that “any material someone provides to another has the potential to be shared online and the difficulty of removing potentially compromising material placed online” and to learn therefore “not to provide material to others that they would not want shared further and not to share personal material which is sent to them” (DfE, 2019, p. 28). It is unlikely that young people would be told not to share anything personal in offline contexts, as personal disclosures are part of relationships. The guidance thus endorses victim-blaming in its phrasing of online risks. Further, pupils are to learn “that sharing and viewing indecent images of children (including those created by children) is a criminal offence which carries severe penalties including jail” (DfE, 2019, p. 28). Such messaging ignores the increasing nuance taken by the police and Crown Prosecution Service to youth-involved intimate image sharing, while, moreover, conflating victim and perpetrator categories in cases of abuse and adult-involved crimes. This negative and problematic messaging may relate to the framing of online cultures as inherently harmful (discussed further below under “programmatic goals”). The underlying principle of the guidance is, therefore, that young people possess rights to sexual health and well-being, but what constitutes health and well-being is framed narrowly and delimits the positive potentialities of sex and relationships by constraining these to “stable” and “committed” relationships and emphasizing the risks of digital media.

### Expansion of Programmatic Goals

Within the guidance, sex, relationships, and sexuality are linked to mental health, well-being, and young people’s ability to thrive in life. The guidance, therefore, goes beyond risk avoidance and recognizes the value of sex and relationships to human health and well-being, suggesting an expansion of programmatic goals. There is emphasis on “positive aspects of healthy one-to-one intimate relationships” (DfE, 2019, p. 29), rather than merely focusing on negative outcomes (except for digital media). Educators are encouraged to provide knowledge and equip young people with the skills to identify and develop healthy relationships and distinguish these from “unhealthy” relationships (sexual/intimate and otherwise). The inclusion of opportunity for skill development within the curriculum evidences an expansion of programmatic goals through aiming to impart “practical steps” (DfE, 2019, p. 21) to improve relationships; practice “strategies for identifying and managing sexual pressure, including understanding peer pressure, resisting pressure and not pressuring others” (DfE, 2019, p. 29); negotiate consent by learning to “actively communicate and recognize consent from others, including sexual consent and how and when consent can be withdrawn” (DfE, 2019, p. 29); recognize prejudice and understand how it can occur; and act upon “responsibilities of bystanders to report bullying and how and where to get help” (DfE, 2019, p. 22). These objectives draw upon and aim to improve assertiveness and civic engagement, moving beyond competencies required for sexual health and romantic intimacy.

Learning objectives also include a wider, deeper cultivation and practice of resilience and character in the individual. RSE is positioned as “…helping to foster pupil wellbeing and develop resilience and character that we know are fundamental to pupils being happy, successful and productive members of society” (DfE, 2019, p. 5). It is to prepare pupils for participation in society and can help them “…achieve goals… [and develop] personal attributes including kindness, integrity, generosity and honesty” (DfE, 2019, p. 5). It is thus evident that a goal of RSE is to help young people integrate into society, but the outlook is traditional or conservative in nature. Young people are to understand “right and wrong” and “ensure [they] take responsibility for their actions” (DfE, 2019, p. 30). It is about socializing young people to contribute to society, with nothing said about how RSE can empower young people to challenge or rework traditional ideas or ways of being.

Young people are, therefore, predominantly positioned as learners rather than agents. They are to absorb facts and information so as to act appropriately and make “good” choices in line with existing societal norms/expectations, with it stating that they are to learn that they can “expect to be treated with respect by others, and that in turn they should show due respect to others, including people in positions of authority and due tolerance of other people’s beliefs” (DfE, 2019, p. 21). The inference is one of civic duty or social responsibility, rather than increasing capacity for civic engagement through criticality, reasoning, and debate. There is the potential for debate about religious teachings on sexuality, but the purpose here seems to be to present facts about different beliefs, rather than to promote critical discussion.

Accompanying some of the more positive goals in the learning outcomes is an emphasis on risk avoidance, for example, the “risks connected to drugs and alcohol” (DfE, 2019, p. 4) and “how the use of alcohol and drugs can lead to risky sexual behaviour” (DfE, 2019, p. 29). The negative framing here decontextualizes RSE from the realities of when individuals have sex and how risk and opportunity are perceived and negotiated. As explored above, digital media is discussed purely in terms of risk. After stating that young people’s lives are digitally mediated and involve a seamless transition between online and offline contexts, the guidance goes on to list risky or problematic behaviors or issues, for example, “…extreme, unkind or exaggerated” behaviors; “websites may share personal data about their users…Individuals can operate online scams” (DfE, 2019, p. 9). There is no corresponding list of the positive aspects of online interaction/relationship and little recognition that digital media can play a positive role in young people’s lives and sociosexual development. The guidance states that “Pupils should know the characteristics of positive and healthy friendships (in all contexts, including online)” (DfE, 2019, p. 17), presupposing that relationships online can be positive and healthy, but the learning outcomes are otherwise negative in tone.

There is also the goal to “protect young people” (DfE, 2019, p. 30) stated within the guidance. Learning outcomes for families include how to determine when people are “trustworthy,” and when relationships are “unsafe” (DfE, 2019, p. 27) and, if the latter, how to seek help. The teaching on healthy relationships also makes a connection to government guidance on Sexual violence and sexual harassment between children in schools and colleges (DfE, 2017) and underscores the protective importance of RSE in preventing violence, abuse, and harassment:

“An understanding for all pupils of healthy relationships, acceptable behaviour and the right of everyone to equal treatment will help ensure that pupils treat each other well and go on to be respectful and kind adults” (DfE, 2019, p. 14).

The teaching of pupils with special educational needs (SEND) is framed predominantly in terms of risk and vulnerability, with the guidance stating, for example, that “[s]chools should be aware that some pupils are more vulnerable to exploitation, bullying and other issues due to the nature of their SEND” (DfE, 2019, p. 15). Overall, there is recognition of the importance of helping young people with SEND to achieve positive experiences of sex and relationships, but this goal is lessened somewhat by the focus on protection and risk.

### Broadened Content

The guidance includes broadened content as it attends to traditionally neglected topics, for example, fertility, menopause, miscarriage, and abortion. It also discusses abusive practices in relationships, families, and communities, for example, grooming, sexual exploitation, domestic abuse (including controlling and coercive behaviors), and female genital mutilation (FGM). Pupils are to be instructed in the law and how to get help if required. There is reference to the specific needs of pupils, for example, that RSE is to be accessible to SEND pupils (DfE, 2019, p. 15). There is, therefore, recognition of current issues connected to sex and relationships and clear commitments to equality and diversity in terms of social justice within young people’s relational contexts, school cultures, and climates.

The framing continues, however, to be legalistic. Abusive, harassing, and bullying behaviors are discussed in terms of empowering young people to report these behaviors and to understand “…the legal rights and responsibilities regarding equality (particularly with reference to the protected characteristics as defined in the Equality Act 2010) and that everyone is unique and equal” (DfE, 2019, p. 28). They are to learn “the concepts of, and laws relating to, sexual consent, sexual exploitation, abuse, grooming, coercion, harassment, rape, domestic abuse, forced marriage, honour-based violence, and FGM, and how these can affect current and future relationships” (DfE, 2019, p. 29). They are also to learn skills in negotiating consent and pressure, but there is no discussion of the realities of and obstacles to implementing these techniques or acting in line with legal understandings in lived contexts.

A decontextualized approach is also evident elsewhere. For example, “facts” about contraception (DfE, 2019, p. 28) are presented as self-evident, with no recognition of the contingencies of and constraints on choice and decision making in lived contexts. There is also perhaps an implicit focus on heterosexual females. While the guidance includes male and female fertility issues, there is a reproductive focus associated with topics such as menstruation, pregnancy, abortion, and menopause despite brief allusion to pleasure. This results in a narrow conceptualization of sex as heterosexual intercourse, ignoring the range and diversity of sexual activities that young people may engage in (Pound et al., 2017). There is little about how to make content meaningful for pupils with more diverse needs along the spectrum of sexuality and gender. For example, there is no acknowledgment of pre-exposure prophylaxis (PREP) or post-exposure prophylaxis (PEP). While not protecting against pregnancy, they protect against HIV transmission and should be acknowledged as such.

Further broadened content includes discussion of different family types and a new emphasis on online behaviors and pornography. The inclusion of different family types is, however, overshadowed by a recurrent emphasis on marriage. Young people are to be taught that while marriage/civil partnerships should be “freely entered into,” there is no recognition of other lifestyle choices and they are to learn that if they are not married or in a civil partnership, then their relationships will not have the same “legal rights and protections” (DfE, 2019, p. 27). The broadening of content to include discussion of online behaviors is also limited as this is the only section where there are no positive statements about this aspect of sex and relationships and no positive outcomes identified for those engaging in these behaviors. Online sexual content is depicted as inherently harmful. For example, pupils are to learn:

“…that specifically sexually explicit material e.g. pornography presents a distorted picture of sexual behaviours, can damage the way people see themselves in relation to others and negatively affect how they behave towards sexual partners” (DfE, 2019, p. 28).

It is evident, therefore, that while the guidance pertains to broaden content, the detail and framing of this content is somewhat limited.

### Youth-Centered Pedagogy

The inclusion of some broadened content suggests a recognition that young people’s sociosexual lives are changing. The guidance advocates that RSE should reflect the realities of sex, sexuality, and relationships for young people, stating that they “should feel that the content is relevant to them and their developing sexuality” (DfE, 2019, p. 26). This is evident through an emphasis on “choice.” Young people are to be made aware of the “choice to delay sex or to enjoy intimacy without sex”; “facts about the full range of contraceptive choices”; and that “there are choices in relation to pregnancy” (DfE, 2019, p. 29), suggesting that they have rights to decision making to shape their sexual health, interactions, and outcomes. While presented as choices, this is, however, somewhat limited by the implication that some behaviors and choices should be avoided (e.g., sex). For example, it is stated that pupils should learn “the reasons for delaying sexual activity” (DfE, 2019, p. 25).

The notion of choice is also extended to schools as the guidance deliberately gives schools “flexibility to shape their curriculum according to the needs of their pupils and school” (DfE, 2019, p. 4). It states that “the policy [that the school develops for RSE] should also reflect the views of teachers and pupils. Listening and responding to the views of young people will strengthen the policy, ensuring that it meets the needs of all pupils” (DfE, 2019, p. 12). This stated commitment to a youth-centered approach is, however, juxtaposed against the right of teachers to decide upon “age-appropriate” content and of faith schools “to reflect on faith teachings about certain topics as well as how their faith institutions may support people in matters of relationships and sex” (DfE, 2019, p. 13). The guidance also upholds, although delimits, parental rights to withdraw. It states that “…parents and carers are the prime educators for children on many of these matters” (DfE, 2019, p. 4) and “are the first educators of their children” (DfE, 2019, p. 17). Parents only have the right to withdraw their child from sex (but not relationships) education, and any request to do so should involve discussion with the school and, “as appropriate,” the child (DfE, 2019, p. 17). Schools are encouraged to speak with parents about the benefits of RSE and work with them to explore the implications of removing their child, including:

“…any social and emotional effects of being excluded, as well as the likelihood of the child hearing their peers’ version of what was said in the classes, rather than what was directly said by the teacher (although the detrimental effects may be mitigated if parents propose to deliver sex education to their child at home instead)” (DfE, 2019, p. 17-18).

These statements limit young people’s rights to learning “correct” information about sex rather than their broader participatory rights. The guidance states that ultimately if a parent wishes to remove their child from sex education, then that is to be honored. It is only once a child turns 16 that they can receive sex education notwithstanding their parents’ wishes (if they express a desire to participate). All this means that teachers and, to a lesser extent, parents can act as gatekeepers of young people’s rights to education.

Parents have “no right to withdraw from Relationships Education or Health Education” (DfE, 2019, p. 18), suggesting a demarcating of sex education which is perhaps deemed more risky, controversial, or personally significant to parents than relationships education. Irrespective of rights to withdraw, schools are to “work closely with parents when planning and delivering these subjects… ensure that parents know what will be taught and when, and clearly communicate the fact that parents have the right to request that their child be withdrawn from some or all of sex education delivered as part of statutory RSE” (DfE, 2019, p. 17). These stipulations make it possible to by-pass learners’ rights. It is also unclear how those in non-mainstream schools are to receive RSE. The guidance applies to “non-maintained special schools, maintained special schools and alternative provision, including pupil referral units” (DfE, 2019, p. 7), but there is no detail about how pupils’ needs are to be met.

The guidance suggests that the content of RSE should be youth-led to some extent, as it advocates for the use of anonymous question boxes to cater teaching to support student needs.

“Knowledge about safer sex and sexual health remains important to ensure that young people are equipped to make safe, informed and healthy choices as they progress through adult life. This should be delivered in a non-judgemental, factual way and allow scope for young people to ask questions in a safe environment. Many teachers use approaches such as distancing techniques, setting ground rules with the class to help manage sensitive discussion and using question boxes to allow pupils to raise issues anonymously” (DfE, 2019, p. 25).

This implies, however, that RSE is an opportunity for adult experts (teachers) to appropriately instruct young people. Following this approach, RSE may seek to be responsive to pupils, but adults are delivering the answers in line with pre-established facts, normative standards for good decision making and fixed ideas of development. Information and knowledge are to be imparted in alignment with a fixed, developmental approach to child/youth sexuality:

“…content must be age appropriate and developmentally appropriate. It must be taught sensitively and inclusively, with respect to the backgrounds and beliefs of pupils and parents while always with the aim of providing pupils with the knowledge they need of the law” (DfE, 2019, p. 4).

This approach has specific implications for LGBT students. While the guidance states that all pupils’ needs should be met, LGBT content is to be taught “at the point at which schools consider it appropriate” (DfE, 2019, p. 15). There is a contradiction in the aim that “all pupils should feel that the content is relevant to them and their developing sexuality” (DfE, 2019, p. 26), which would appear inclusive and youth-focused while isolating these students and relegating their needs by recommending that “sexual orientation and gender identity should be explored at a timely point” (DfE, 2019, p. 15).

The scope for youth-centered pedagogy afforded by the guidance is, therefore, limited. There is recognition of young people’s changing lives and the need to connect with the realities of their developing sociosexual subjectivities, but the guidance takes a “teacher-expert” standpoint and is preoccupied with facts-based knowledge building designed to guide development and decision making in line with normative standards and expectations.

## Discussion

This analysis examined the extent to which and how the DfE (2019) statutory guidance on RSE for schools in England upholds young people’s rights to receive RSE, their reciprocal rights to positive sexual health and well-being, and a rights-based pedagogy based on active and participatory learning. The analysis suggests that rights are, to some extent, advanced within the guidance. The guidance includes an attempt to teach key skills, in line with evidence on best practice in RSE that underscores the importance of skills development (Pound et al., 2017). However, it contains explicit and implicit heteronormativity and takes a negative, risk-averse, and harm reduction approach to aspects of young people’s sociosexual development, particularly digital media and issues deemed to be specific to the “LGBT community.” It advances abstract, legalistic, and decontextualized notions of reciprocal rights to sexual health and well-being, designed to support informed choice and decision making, echoing the findings of others regarding how the focus of RSE policy and practice is often on safety, risk, and the law to the detriment of pleasure and ethics (Bragg et al., 2021; Carmody, 2015; Whittington & Thomson, 2018). Therefore, the extent to which the stated commitment to positive sexual health and well-being will be realized through the learning outcomes may be limited. This decontextualized, risk-averse, and legalistic approach is disengaging to learners who want to learn skills that will help them avoid risk and to “become more confident in sexual negotiations” (Pound et al., 2017, p. 4; Whittington, 2020).

One of the biggest features of contemporary youth sexual and relational culture—digital media—is framed entirely outside of positive sexual health and well-being in a risk-averse and negative way. The guidance is negative when discussing online behaviors. It raises the impact of viewing harmful content but provides no description of what this impact is, perhaps because the evidence base for these claims is not always robust (Marston, 2018). It also simplifies and stigmatizes young people’s varying experiences (positive and negative) with sexualized digital media. Such media can, for example, be helpful for LGBT youth whose perspectives are often marginalized or, as seen within the learning outcomes, relegated within mainstream RSE (DeHaan et al., 2013; Jenzen, 2017; McGeeney & Hanson, 2017). There is also little acknowledgment of digital relationships with family and friends, despite evidence that online interactions give young people a sense of belonging and make friendships visible in spaces that are perceived as relatively safe (Attwood, 2017; Waite, 2011).

In upholding a legalistic and facts-based approach to RSE that honors young people’s rights to information and knowledge to support choice and decision making, the guidance aims to prepare young people for participation in a democratic society as reasoned and productive individuals. The emphasis on legality serves, however, to remind young people that while they can be protected by the law, they can also be punished. Furthermore, the decontextualized framing and the implicit value judgments about choices and lifestyles marginalizes particular young people. The guidance is largely focused on heteronormative relationships—monogamy and marriage—while other relationships are ignored; civil partnerships are mentioned but relegated to a footnote. The guidance alludes to the “characteristics and legal status of *other* types of long-term relationships” (DfE 2019, p. 27, emphasis added), suggesting an othering of non-traditional relationships and a lack of acknowledgment of non-long-term relationships. There is no recognition of safe-sex methods to protect against STIs and HIV outside the boundaries of heteronormative contraceptive approaches to prevent pregnancy (e.g., no reference to PREP, PEP, or dental dams), perhaps because this conflicts with the implicit values evident within the guidance.

The normative judgments and value systems evident within the guidance may be intended to pre-empt and defend against accusations that liberal RSE will encourage “undesirable” behaviors or will corrupt young people, and, therefore, any backlash from parents, faith groups or others. For example, the guidance emphasizes that:

“Effective RSE does not encourage early sexual experimentation. It should teach young people to understand human sexuality and to respect themselves and others. It enables young people to mature, build their confidence and self-esteem and understand the reasons for delaying sexual activity. Effective RSE also supports people, throughout life, to develop safe, fulfilling and healthy sexual relationships, at the appropriate time” (DfE, 2019, p. 25).

This statement implies that RSE can promote “correct” choices and behaviors and reduce risk, with the positive potentialities of sex and relationships to come later on at the “appropriate time.” This is particularly notably regarding LGBT topics, which have long been marginalized within RSE (Pound et al., 2017). These topics are to be introduced at “the point at which schools consider it appropriate” (DfE, 2019, p. 15), suggesting that heterosexuality and heteronormativity will otherwise be prominent. LGBT topics are, essentially, to remain taboo or secret until some unspecified time, while, presumably, heterosexual relationships are safe and acceptable at any age. This perhaps illustrates how young people’s rights can be delimited in practice due to how the guidance balances their rights with parental rights and teacher flexibility and discretion.

There is acknowledgment that RSE can, and should, be part of a whole school approach to promoting equality and diversity. However, there is limited explicit or specific translation of equality and justice concerns within the learning outcomes. Any sociocultural context of inequality and injustice is deemphasized in favor of a legalistic framing of rights. The rights in the guidance are individualistic, decontextualized, and advance normative standards around sex, sexuality, and relationships that are conservative and restrictive in nature. There is limited recognition of the realities of how rights are enacted and upheld (or not) in lived contexts nor of how the operation of rights is socially contingent and structurally constrained (see Attwood, 2006; Fine & McClelland, 2006; Mayo, 2011; Tolman, 2012). Thus, there is no evidence that RSE holds potential for emancipatory change. While there is some reference to young people’s lives and sociosexual subjectivities and self-concepts, such recognition appears to be in service of a traditional “adult expert” model of RSE rather than a reciprocal model of learning involving active pupil participation. There is, therefore, a missed opportunity to frame rights as relational and to engage with the social and cultural contingencies and contexts that shape young people’s developing sexual subjectivities and experiences. For example, the emphasis on teaching the law around sexual consent “reinforce[s] legalistic and binary notions of consent/rape which do not map onto young people’s experiences of navigating sex and relationships” (Whittington, 2020, 480).

The guidance thus claims to be supporting young people’s rights to RSE and upholding equality and diversity, while advancing conservative principles and leaving schools responsible for joining the dots in practice but will little specific support to do so.

### Implications for Practice

The findings from the analysis of the guidance have implications for how RSE policy may be implemented and enacted in schools in England. For example, the discretion given to schools in deciding at which point they consider it appropriate to teach about LGBT-related topics suggests that LGBT students may not get equitable access to appropriate and timely RSE across the country, meaning that the issues with inconsistency in provision may continue with particular impacts on already-marginalized groups. As another example, the presentation of “healthy relationships” as solely monogamous means that youth who are engaging with multiple partners may not get access to important information about how to protect and enhance their sexual health when in sexual relationships with multiple people. As such, we recommend that the guidance is taken as a *minimum* standard of rights and that those responsible for policy implementation and enactment within schools develop their local policy and curriculum in ways that ensure equitable access to full and holistic education for all pupils. As elaborated upon below, this requires a collaborative and participatory approach to designing and delivering the RSE curriculum with pupils.

The RSE curriculum has evolved in response to political pressures, media-driven moral panics, and public perceptions (Carmody, 2015; Gilbert, 2018). By representing more of a conceptual definition of RSE, Berglas et al. (2014b) principles of rights-based RSE require operationalization into actionable objectives before they can be translated into practice. Firstly, when delivering RSE, educators should be mindful that young people are legitimate sexual agents. Alongside the set curriculum, pupils should have the opportunity to identify their own topics for discussion, which may increase the likelihood that they will feel able to contribute their own perspective on the subject content in a meaningful way. Adopting such an approach should produce RSE that reflects young people’s lived experiences and is inclusive of subjective situated realities. We acknowledge that given that educators act in loco parentis, it may be a challenge for them to do this, but we would suggest that adopting a different mindset would help RSE to move beyond the set curriculum and to develop a more youth-centered and responsive approach.

To establish this culture, consultation might start with issues that can be resolved along the lines proposed by pupils, to build confidence in the process (Bragg, 2010). In RSE, this could work by educators asking, for example, when RSE should be delivered and how. When a program of RSE has been decided, pupils should be consulted again to determine if there are any topics that that have concerns about and how to address these and/or if there are any favored or less favored delivery styles (e.g., mixed sex lessons, teacher-delivered lectures, etc.). There is an extensive array of possible methods for eliciting pupils’ perspectives ranging from suggestion boxes, graffiti walls, “statement trees,” and voting systems to facilitate anonymous contributions, to in-person debate and discussion, along with visual prompts and journey maps to illustrate and remind pupils and educators about their roles and how the curriculum builds over time. There are toolkits available with practical solutions, for example, “Are You Getting It Right?” developed based on research with secondary school pupils (Martinez & de Meza, 2008). Practical suggestions include using cards to prioritize and suggest different RSE topics. Teachers could also ask pupils to record things that “jar” them about RSE which could then be explored and discussed as a class to develop solutions (Renold, 2016).

Youth-centered approaches to identifying and prioritizing what is of relevance and interest will not, however, necessarily be solutions in and of themselves. Mayo (2011) questions who gets to define what is relevant and of interest; referring to Ferree (2003), she suggests that educational messages may need to be radical rather than just what resonate because the latter can be conservative. Whittington (2020, p. 3) advocates acknowledging the gray areas of RSE and utilizing “the device of the continuum” in order to “encourage processual and ethical thinking” in RSE. She argues that “… practicing continuum thinking offers opportunities for critical teaching and learning by considering context in the form of the environmental, material, ethical, and relational aspects of an encounter and the perceived agency and competence of those involved” (Whittington, 2020, p. 10). Regardless, for young people to set the agenda, their suggestions, even when divergent from the objectives in government guidance, should be included or at least acknowledged. This may be challenging given ongoing debates around what is “appropriate” for young people to learn and discuss.

Educators should also consider that content needs to be inclusive of pupils and parents with different protected characteristics. While the guidance may avoid gendered or heteronormative phrasing, this does not mean that problematic practices are no longer an issue, for example, problematizing youth sexuality (either in general or regarding specific types of young people). This necessitates an acknowledgment of pleasure to create a positive, supportive arena for sexual development. Doing so will be difficult in a context in which educators do not want to be seen as condoning experimentation. Rights-based RSE is, therefore, inevitably challenging to enact in practice.

These challenges are not just value-based but also relate to other demands on the school day and the need for schools to consider the contexts and contingencies of the school, pupils, and wider community, as well as how educators feel about delivering RSE and the type of RSE they feel comfortable delivering (Blake & Aggleton, 2017; Goldman, 2012). Youth digital intimacies, due to its associated legal issues, may be particularly challenging to openly address*.* The emphasis on harms and protection exacerbates the “policing versus promoting” dilemma currently experienced by educators. Yet, digital sexualities for youth are addressed in various international rights frameworks that uphold both protection from harm and freedom of expression (Crofts & Lievens, 2018), and it is unlikely that education on digital intimacies will be meaningful or effective until a more participatory and less risk-averse approach is adopted (see Lee et al., 2018).

On a practical level, there is a question over whether schools and educators are adequately equipped (in terms of training and resources) to deliver RSE and whether RSE can acquire status and esteem within schools to improve the relationship between RSE and the wider school curriculum (Abbott et al., 2015; Spencer et al., 2008). It is also important to consider the wider pedagogical paradigms within which schools and educators are operating. Spencer et al. (2008) argue that there is a conflict between pupil-centered approaches and the emphasis on skills and learning outcomes in school curricula (particularly when there is a lack of straightforward answers or “facts” around taught topics). Pupil-led, rights-based RSE may also be difficult in contexts of protectionism over young people, lack of time for RSE in the curriculum, and limitations on the extent to which young people are able to identify the issues that are important to them if they have limited sexual experience and/or limited ability to articulate their needs.

Attention also needs to be given, as the guidance states, to contexts beyond the classroom. Schools are involved in the sexual socialization of youth beyond formal RSE classes, and “anti-oppressive” education would be mindful of how oppressive structures are located within the wider school curriculum, school policies and procedures, and the school climate (Connell & Elliot, 2009; Elia & Eliason, 2010; Jones, 2011). There is a “hidden curriculum” driven by the language and behavior of school staff, how they react to and frame incidents and lessons to students, their interactions with students, and so on. This hidden curriculum can be heteronormative and sexist, if, for example, there are different expectations based on gender and sexuality, and what goes accepted and uncommented upon. Class and race also intersect here in terms of expectations and treatment.

The “ignored curriculum”—what young people learn from one another in their peer cultures—is also important (Gougeon, 2009). RSE needs to be grounded within these lived experiences and ongoing realities, rather than tackling issues as discrete or conceiving of these cultures as inherently problematic. As such, Spencer et al., (2008, p. 350) contend that it is important to interrogate “…the ‘places’ in which young people’s sexual relationships and experiences are negotiated as well as the relations of power within these different contexts.” These may be structural, for example, relating to gender or sexual identity, or may be institutional, for example, in terms of access to information or services. It is hoped that a more contextualized approach means that tensions between parental rights or cultural concerns and young people’s rights can be deemphasized in favor of RSE that acknowledges the history, culture, and other places from which values emerge (Lamb, 2010), in which sexuality and sexual rights are conceived of as relational and interconnected (Cense, 2019; Kenneally, 2017; Midema et al., 2020; Rasmussen, 2012). In this sense, rights-based RSE is both about how young people learn about and enact their sexual subjectivities within their lived contexts and how they are positioned as learners within RSE itself regarding the extent to which they can actively engage with the pedagogical process and have recognized their diverse lived experiences and situated realities (see Davies & Kenneally, 2020).

From this perspective, RSE can become about “[c]apturing… instances of resistance and engaging young people in a discussion of alternative discourses, [which] may facilitate consciousness raising of a form that enables both young people and those ‘running’ the school to open up new opportunities for discussing young people’s sexuality” (Spencer et al., 2008, p.353). This approach involves engaging with the position of young people within the pedagogic process; they need to be seen as autonomous agents, rather than following the fixed developmental trajectories as suggested in RSE guidance, with education imparted at “teachable moments” within and beyond formal RSE lessons (Elia & Eliason, 2010, p. 44).

### Future Research Agenda

There is a limited evidence base to aid the design and delivery of the rollout of mandatory RSE. Interventions that have evaluated rights-based approaches to RSE delivered in secondary schools report positive changes among participants (Constantine et al., 2015; Jewkes et al., 2008; Rijsdijk et al., 2014; Rogow et al., 2013; Rohrbach et al., 2015). Constantine et al. (2015) identified improved knowledge and attitudes, while Rohrbach et al. (2015) report that rights-based pupils scored higher than control group of basic RSE pupils on sexual health knowledge, attitudes about relationship rights, partner communication, protection, self-efficacy, access to health information, and awareness of sexual health services. This evidence also suggests, however, that a rights-based approach may not be as effective at changing behavior (Constantine et al., 2015; Jewkes et al., 2008; Rohrbach et al., 2015). While this evidence portrays rights-based RSE in a positive light, research literature is subject to several limitations.

Firstly, the lack of consensus around the definition of rights-based RSE has led to an application of the label to a highly divergent range of programs. Within the literature, many interventions fall under the label of rights-based programs and are used by others as evidence of rights-based success, but interventions often focus on gender equity rather than a broader concept of sexual rights (e.g., Pulerwitz et al., 2010; Rogow & Haberland, 2005). Even where programs appear to be conceptually similar, it is unclear whether they are delivering the same message or testing the same construct. This echoes similar observations made by Berglas et al. (2014b), p. 289) who noted that “there has been limited empirical research undertaken to address questions of program design or effectiveness.”

Furthermore, while these studies may be used to support a rights-based approach to RSE, it is questionable to what extent it is appropriate to make judgments about effectiveness of rights-based RSE program in English secondary schools based upon existing research evidence. Studies typically take place within the community rather than school-based programs (Constantine et al., 2015; DiClemente et al., 2014; Jewkes et al., 2008; Rogow & Haberland, 2005), are outside of UK (e.g., Crepaz et al., 2009; Marques & Ressa, 2013; Rohrbach et al., 2015; Rijsdijk et al., 2014; Rogow et al., 2013) and are case studies or process evaluations, providing no comparative data for existing alternatives (e.g., Marques & Ressa, 2013; Rijsdijk et al., 2014; Rogow et al., 2013). It is therefore difficult to determine to what extent rights-based RSE can lead to improved attitudes or behavior compared with alternative approaches. In addition, studies have not been subject to methodological or conceptual appraisal via systematic review (Berglas et al., 2014a), preventing compilation and evaluation of the breadth and rigor of the existing evidence base.

As a result, existing interventions should be subject to systematic review to determine the “state of the art.” Due to the identified limitations with existing research, it would also be beneficial to undertake a controlled trial of a rights-based intervention in UK secondary schools. Topics within the statutory curriculum could be delivered in different styles, with rights-based RSE compared against a control group of pupils receiving material delivered using a risk-avoidance approach. Given the solely negative framing of young people’s digital lives, it is particularly pertinent that a rights-based approach to digital intimacies is examined to identify how education on this topic can more accurately reflect lived realities and experiences. Further research will be required to explore how pupils appraise the applicability of rights-based approaches to their own relationships and how teachers can apply these principles in practice as presently, “[n]o systematic research has yet been undertaken to gauge youth perspectives on such a framework of individual rights and responsibilities to determine its applicability or resonance with youth targeted by rights-based sexuality education programs” (Berglas et al., 2014a, p. 289).

## Conclusion

Overall, while the inclusion of equality and diversity issues within RSE guidance suggests an expansion of programmatic goals, learning outcomes do not seem to inspire a critical model of rights-based RSE. Given the focus on “normality,” heteronormativity, and legality, it is unclear how schools will be able to design or deliver “anti-oppressive” RSE following the learning outcomes included in the statutory guidance. Instead, schools are to focus on teaching narrow definitions of equality and diversity as defined by law. While there is an acknowledgment of the right of youth to choose regarding their sexual health and well-being within the guidance, and some advancement of the reciprocal nature of these rights, these are presented through decontextualized notions of reciprocal rights that do not engage with the relational and sociocultural contexts in which rights unfold and agentic decision making and choice occurs (or is constrained).

Through identifying the limitations of the guidance in terms of rights-based RSE, we intend for the analysis presented in this paper to support those responsible for policy implementation and enactment within schools to fill the gaps when designing and developing a local RSE policy and curriculum that addresses pupils’ rights in the broadest and most inclusive sense. The analysis was undertaken systematically in respect to Berglas et al. (2014a) framework; however, it is noted that the interpretations and conclusions presented here are subjective. It is not possible to identify the intentions or objectives of those responsible for drafting the policy, the constraints surrounding the policy making process, or the ways in which the policy is being implemented and enacted within schools. A study involving empirical data collection from policy and practice actors and stakeholders would be required to explore these points. Such a study would identify the top-down factors shaping the policy making process and the bottom-up factors shaping the implementation and enactment process, and, in turn, the conditions required for the development of rights-based RSE policy and curriculum.
